# Structural and Hydrodynamic Characterization of Dimeric Human Oligoadenylate Synthetase 2

**DOI:** 10.1016/j.bpj.2020.04.025

**Published:** 2020-05-01

**Authors:** Amit Koul, Danielle Gemmill, Nikhat Lubna, Markus Meier, Natalie Krahn, Evan P. Booy, Jörg Stetefeld, Trushar R. Patel, Sean A. McKenna

**Affiliations:** 1 Department of Chemistry, University of Manitoba, Winnipeg, Manitoba, Canada; 2 Alberta RNA Research and Training Institute, Department of Chemistry and Biochemistry, University of Lethbridge, Lethbridge, Alberta, Canada; 3 Department of Molecular Biology and Biochemistry, Yale University, New Haven, Connecticut; 4 Department of Microbiology, Immunology and Infectious Disease, Cumming School of Medicine, University of Calgary, Northwest Calgary, Alberta, Canada; 5 Li Ka Shing Institute of Virology and Discovery Lab, University of Alberta, Edmonton, Alberta, Canada; 6 Department of Biochemistry and Medical Genetics, University of Manitoba, Winnipeg, Manitoba, Canada

## Abstract

Oligoadenylate synthetases (OASs) are a family of interferon-inducible enzymes that require double-stranded RNA (dsRNA) as a cofactor. Upon binding dsRNA, OAS undergoes a conformational change and is activated to polymerize ATP into 2′-5′-oligoadenylate chains. The OAS family consists of several isozymes, with unique domain organizations to potentially interact with dsRNA of variable length, providing diversity in viral RNA recognition. In addition, oligomerization of OAS isozymes, potentially OAS1 and OAS2, is hypothesized to be important for 2′-5′-oligoadenylate chain building. In this study, we present the solution conformation of dimeric human OAS2 using an integrated approach involving small-angle x-ray scattering, analytical ultracentrifugation, and dynamic light scattering techniques. We also demonstrate OAS2 dimerization using immunoprecipitation approaches in human cells. Whereas mutation of a key active-site aspartic acid residue prevents OAS2 activity, a C-terminal mutation previously hypothesized to disrupt OAS self-association had only a minor effect on OAS2 activity. Finally, we also present the solution structure of OAS1 monomer and dimer, comparing their hydrodynamic properties with OAS2. In summary, our work presents the first, to our knowledge, dimeric structural models of OAS2 that enhance our understanding of the oligomerization and catalytic function of OAS enzymes.

## Significance

Human 2′-5′-oligoadenylate synthetases (OASs) are a family of interferon-inducible enzymes that form an important component of the innate immune system. The OAS family consists of different OAS enzymes: OAS1, OAS2, and OAS3. OAS enzymes are activated upon interaction with viral double-stranded RNAs and synthesize 2′-5′ linked oligoadenylate chains from ATP that further activate ribonuclease L, ultimately restricting viral propagation. We present the first, to our knowledge, low-resolution structural model of OAS2 dimer and confirm dimer formation in a cellular background. Our work provides insights into the recognition of double-stranded RNA of different lengths by OAS enzymes, critical for the innate immune response.

## Introduction

The 2′-5′-oligoadenylate synthetases (OASs) are interferon-induced proteins that catalyze the synthesis of 2′-5′-linked oligomers (2-5A) upon interaction with double-stranded RNA (dsRNA) and are often triggered by viral infection (1). 2-5A can activate latent ribonuclease L by causing its dimerization (2), which leads to the degradation of viral and cellular RNAs (3,4). This degradation limits protein synthesis, thereby impairing viral replication (5,6). In humans, there are three catalytically active members in the OAS family; OAS1, OAS2, and OAS3 (7,8). OAS1, OAS2, and OAS3 consist of one, two, and three domains, respectively; however, only one domain is catalytically active (9). The OAS catalytic domain shares similarity with polymerase *β* (10). Three aspartic acid residues in the active site form the core catalytic triad responsible for the coordination of two magnesium ions and are required for both the binding of ATP and 2-5A chain formation (11). Mutation of these residues to alanine abrogates OAS activity (12). In OAS1, the triad is present at positions D75, D77, and D148 (13). OAS2 consists of two OAS1-like domains (domains I and II), but only domain II contains the catalytic aspartic acid residues at positions D408, D410, and D481 (14). OAS3 has three OAS1-like domains (domains I, II, and III), with domain III harboring the catalytic triad at positions D816, D818, and D888 (15).

OAS enzymes are present in most mammalian cells and tissues (16,17) and have been shown to provide protection against various viruses. For example, OAS1 provides resistance against picornavirus, dengue, and Japanese encephalitis virus (18, 19, 20), whereas OAS2 is effective against encephalomyocarditis virus and porcine reproductive and respiratory syndrome (21,22). A single nucleotide polymorphism in OAS3 abolishes antiviral resistance against chikungunya virus (23). Additionally, OAS enzymes are involved in cell growth, differentiation, and apoptosis (24, 25, 26).

dsRNA interacts with a channel of positively charged residues on the face opposite to the OAS active site (13,27). OAS1 contains two dsRNA binding sites located ∼30 Å apart, creating a surface that interacts with two consecutive minor groves in the dsRNA and establishing a minimum length requirement of ∼17 bp (13). OAS2 is activated by ∼35 bp dsRNA, whereas OAS3 has a preference for ∼50 bp dsRNA (13,28,29). The RNA-protein interactions are mediated in large part by the 2′ hydroxyl groups on the dsRNA (27). Upon interaction with dsRNA, OAS enzymes undergo a conformational change that moves the aspartic acid residues in the active site into close proximity, allowing the coordination of two magnesium ions required for binding of ATP that ultimately leads to the catalysis of 2-5A chains (13,30).

Oligomerization of OAS enzymes and/or the orientation of individual OAS domains has been hypothesized to play a role in enzymatic activity. OAS1 forms monomer, dimers, and tetramers (31), whereas OAS2 can form a dimer (28,32). OAS3 is not known to oligomerize (15). Although bacterial OAS1 expression systems are well established and have led to high-resolution OAS1 crystal structures (13,27), there is no full-length, high-resolution structure available for OAS2 or OAS3. The low-resolution structure of OAS3 suggests an extended structure in solution (15). Three amino acid residues (C331, F332, and K333) are present at the C-terminus of OAS1 or in domain II of OAS2 (C668, F669, and K670) that are thought to potentially mediate oligomerization (14,31). However, this motif is absent in OAS3, which could account for its lack of oligomerization (12).

Although the high-resolution structures of OAS1 and domain I of OAS3 have provided important insights into the recognition of dsRNA, the importance of OAS self-association has not been as extensively studied (13,27,29). Studies of OAS2 structure have been limited, partly because of protein expression and aggregation issues, low protein yields, and lack of homogeneity in OAS2 purification (32). We have previously reported a detailed protocol for homogenous preparation of OAS2 in eukaryotic cells (28). In this study, we characterized the solution properties of recombinant human OAS2 using an integrated set of biophysical methods, including size-exclusion chromatography linked with small-angle x-ray scattering (SEC-SAXS), analytical ultracentrifugation (AUC), dynamic light scattering (DLS), and circular dichroism (CD). Our data suggest that OAS2 forms a dimer in solution comprising four OAS domains (with two domains contributed from each OAS protomer). Coimmunoprecipitation assays in human cells demonstrated that a high-affinity dimer can be observed. We further explored oligomerization behavior and catalytic activity by mutational studies. Mutation of the catalytic aspartic acid residue D408 to A408 at the catalytic triad leads to abrogation of OA2 activity. We also investigated the solution properties of recombinant human OAS1 using biophysical methods, which suggests that OAS1 can exist as monomer and dimer in solution, but not as a tetramer, under the experimental conditions employed (31).

## Materials and Methods

### Expression and purification of OAS2 and its mutants

Protein expression in HEK293T cells and subsequent purification of FLAG-tagged OAS2 (FLAG-OAS2) and His-tagged OAS2 (HIS-OAS2) were performed as described previously (28), with the exception of HIS-OAS2 primers that code for an N-terminal HIS-tag being used. FLAG-tagged CAFAKA (a mutation of C668, F669, and K670 to alanine) was designed by amplifying it from a pcDNA3 vector carrying a wild-type OAS2 by PCR using 1) a forward primer 5′-ATAATTAAGCTTGCCACCATGGACTACAAAGACGATGACGACAAGGGAAATGGGGAGTCCCAG-3′ that had a Hind III restriction site and encodes an N-terminal FLAG (DYKDDDDK) tag and 2) a 100 nucleotide reverse primer 5′-ATCCTACTCGAGTTAGATGACTTTT ACCGGCACTTTCCAAGGTGGTATTGGGTTTCCAGTCCCATCCGCGGCGGCGGGAGAGATAACCATTCCTTTGCT-3′ that contains an XhoI restriction site and converts the codons for C668, F669, and K670 to alanines. The resulting PCR product was digested with XhoI and *Hin*dIII, purified using a Genejet PCR purification kit (Thermo Fisher Scientific, Ottawa, ON, Canada), and ligated using T4 DNA ligase (Thermo Fisher Scientific) into predigested pCDNA3 vector. pCDNA3 vector containing CAFAKA was transformed into NEB Turbo competent *E. coli* (New England BioLabs, Toronto, ON, Canada) using the manufacturer’s protocol. Maxi-Preps (Thermo Fisher Scientific) were performed to obtain plasmid DNA.

Catalytically inactive aspartic acid mutant D481A (in which the aspartic acid at position 481 was mutated to alanine) was constructed by PCR from FLAG-OAS2 in pCDNA3 using an overlapping forward primer (5′-TCCAAAGTCCTCAACGAAAGTGTCAGCTTTGCCGTGCTTCC TGCCTTTAATGCACTGGGTCAG-3′) and reverse primer (5′-CTGACCCAGTGCATTAAAGGCAGG AAGCACGGCAAAGCTGACACTTTCGTTGAGGACTTTGGA-3′). After PCR, the parent plasmid was digested with DpnI (Thermo Fisher Scientific) for 30 min at 37°C in the water bath. Cleanup of the PCR product and isolation of plasmid DNA were performed as described for CAFAKA. All plasmids were sequenced before transfecting HEK293T cells for protein expression. The expression and purification of the OAS2 mutants CAFAKA and D481A were performed as described previously (28).

### SEC

Affinity-purified protein was subjected to SEC using a Superdex 200 10/300 GL gel filtration column (10 × 300 mm; GE Healthcare Life Sciences, Pittsburgh, PA) in 20 mM HEPES (pH 7.2), 300 mM NaCl, 2 mM dithiothreitol (DTT), 0.1 mM EDTA, and 10% (v/v) glycerol. The eluted fractions were monitored by absorbance at 280 nm. Multiple fractions of individual peaks were combined and concentrated using Millipore concentrator filters (Millipore, Burlington, MA). Protein purity was confirmed by sodium dodecyl sufate-polyacrylamide gel electrophoresis (SDS-PAGE), and protein concentration was determined spectrophotometrically by absorbance at 280 nm using the extinction coefficient (129,830 M^−1^ cm^−1^) calculated with the ProtParam tool on ExPASy servers (33).

### OAS enzyme activity assay

OAS activity was measured using an established colorimetric assay that quantifies the amount of pyrophosphate (PP_i_) produced by the catalysis of ATP by 2-5A formation as described previously (28,34), with the exception that 200 nM protein was used for the work in this manuscript.

### DLS

DLS measurements were performed at 20°C on a Nano-S DLS system (Malvern Instruments, Worcester, UK) as described previously (34,35). Before data collection, samples were filtered using a 0.1 *μ*m syringe filter (Millipore). OAS2 was solubilized in a buffer containing 20 mM HEPES (pH 7.2), 300 mM NaCl, 2 mM DTT, 0.1 mM EDTA, and 10% (v/v) glycerol, whereas OAS1 was solubilized in buffer containing 20 mM Tris (pH 7.5), 100 mM NaCl, 1 mM DTT, and 10% (v/v) glycerol. 16 measurements were collected at each protein concentration examined, and the obtained hydrodynamic radii were extrapolated to infinite dilution to yield *r*^0^_*H*_.

### Analytical ultracentrifugation

Sedimentation velocity (SV) data were collected using a ProteomeLab XL-I analytical ultracentrifuge (Beckman Coulter Canada, Mississauga, ON, Canada) with an An-50 Ti 8-place rotor, using absorbance optics at a rotor speeds of 30,000 and 25,000 revolutions per minute (rpm) for OAS2 and OAS1, respectively. We measured OAS2 concentrations of 0.16, 0.41, 0.57, and 1.33 g/L (2.0–16.70 *μ*M) at a wavelength of 294 nm, and OAS1 concentrations of 0.5, 1.0, 1.5, 2.0, 2.5, 3.0, and 3.5 g/L (12–84.13 *μ*M) for OAS1 at 20°C at a wavelength of 297 nm. Before data collection, OAS2 was dialyzed in 20 mM HEPES (pH 7.2), 300 mM NaCl, 0.1 mM EDTA, 2 mM DTT, and 10% (v/v) glycerol, and OAS1 was dialyzed in 20 mM Tris (pH 7.2), 100 mM NaCl, 1 mM DTT, and 10% (v/v) glycerol. 400 *μ*L of each sample was loaded into a channel of the double-sector centerpiece. The rotor with samples were equilibrated to 20°C for ∼2 h under vacuum. Radial scans for OAS2 and OAS1 were collected every 10 and 11 min, respectively. One-dimensional distributions *c*(*s*) of the sedimentation coefficient (*S*) were calculated in *SEDFIT* (36) as shown earlier (37). We then calculated the apparent sedimentation coefficient (*s*), apparent molecular mass (*M*), and oligomerization (if any) at each concentration in *SEDFIT* (36) and converted to standard conditions (pure water at 20°C) with a buffer density (*ρ*_*T*, *b*_) of 1.040600 g/cm^3^ and 1.003800 g/cm^3^, a buffer viscosity (*η*_*T*, *b*_) of 0.014145 P and 0.010270 P, and partial specific volume ${\bar{\nu }}_{T,b}$ = ${\bar{\nu }}_{20{}^{\circ}C,w}$ = 0.74121 and 0.74189 cm^3^/g for OAS2 and OAS1, respectively, to obtain *s*^0^_20°*C*, *w*_ and *M*^0^ (Table 1) for FLAG-tagged OAS2 and OAS1, respectively (38,39).

$$s_{20{}^{\circ}C,w}=s_{T,b}\frac{\eta_{T,b}}{\eta_{20{}^{\circ}C,w}}\frac{\left(1-{\bar{\nu }}_{20{}^{\circ}C,w}\rho_{20{}^{\circ}C,w}\right)}{\left(1-{\bar{\nu }}_{T,b}\rho_{T,b}\right)}.$$

**Table 1:** Experimental and Predicted Hydrodynamic Parameters for Recombinant Wild-Type OAS2

Guinier Data	
*r*_*G*_ (Å)	42.00 ± 0.25
*I*_0_	0.021 ± 7.8 × 10^5^
*q* × *r*_*G*_ range	0.51–1.26
Data points	22–103

**Real-space data**	

*r*_*G*_ (Å)	40.00 ± 0.06
Dmax (Å)	110
*I*_0_	0.01980 ± 4.4 × 10^5^
*q* range (Å^−1^)	0.0120–0.206
Sequence *M*_*w*_ (kDa)	78.78
*M*_*w*_ calculated (kDa) DAMMIN	∼165, dimer
s_20, w_ (S)	4.9 ± 0.3
*r*_*H*_ (nm)	7.5 ± 0.2
*χ*^2^ DAMMIN	∼0.77
NSD DAMMIN	0.55 ± 0.02
*χ*^2^ CORAL	∼0.6
NSD CORAL	2.4 ± 0.11

Graphical representations of the residuals and fits to the SV scans, as well as the *c*(*s*) distributions, were generated with *GUSSI* (40). The density of viscosity of HEPES and Tris buffers were calculated using *SEDNTERP 2* (41).

### CD

For CD experiments, OAS2 was dialyzed in 20 mM sodium phosphate buffer (pH 7.2), 300 mM sodium fluoride, 0.1 mM EDTA, and 10% (v/v) glycerol, followed by data collection using a J-810 spectropolarimeter (JASCO, Easton, MD), and a 0.05 cm quartz cell (Hellma Optik, Jena, Germany). Samples and baseline buffer spectra were measured under a continuous scanning mode with a 2 nm data pitch and a scan speed of 5 nm/min with a response time of 2 s. The CD spectra presented are from the accumulation of four scans of OAS2 protein at 0.478 g/L with the buffer signal subtracted. CD data were deconvoluted using the online DICHROWEB webserver analysis tool (42), which provided a calculated secondary structure profile by comparing the calculated structures and experimental data by CDSSTR and CONTIN-LL (Provencher and Glockner method) using the reference data set 4 (optimized for 190–240 nm) (43). The results are shown in terms of six classification outputs: regular *α*-helix (helix-1)_,_ distorted *α*-helix (helix-2), regular *β* strands (strand-1), distorted *β* strands (strand-2), turns (T), and unordered (U), devised by Sreerama et al. (44). CD classification using mathematical indices splits *α*-helix and *β*-strands into two classes by considering an average four residues per *α*-helix and two residues per *β*-strand to be distorted. In addition, the PHYRE^2^ (45) online webserver analysis of secondary structure and disorder prediction produced consistent protein folding values in normal modeling mode, based on the OAS2 amino acid sequence.

### SAXS

SAXS data for OAS1 and OAS2 samples were collected at 4.14 and 4.00 g/L concentrations, respectively, at the Diamond Light Source (B21 beamline; Didcot, UK) using an SEC-SAXS setup. 50 *μ*L of purified samples was loaded onto a Shodex KW404 column (Shodex, Tokyo, Japan), followed by SAXS data collection every 3 s as described previously (46). An in-line Agilent 1200 (Agilent Technologies, Stockport, UK) chromatography unit connected to a specialized flow cell was used. Appropriate frames from each sample peak region were integrated and buffer subtracted using ScÅtter as described previously (46). The buffer subtracted data were merged using the PRIMUS package from the ATSAS Suite (47), as described previously (48,49).

Next, a Guinier analysis was performed on merged data to obtain the radius of gyration (*r*_*G*_) and study OAS2 homogeneity (50). We also performed dimensionless Kratky analysis to investigate whether OAS2 is folded (51). The *r*_*G*_ and maximal particle dimension (*D*_*max*_) were determined by calculating the pair-distance distribution *P*(*r*) function using the program GNOM (52). The pair-distance distribution information was also used to calculate multiple low-resolution structures using DAMMIN (53), followed by averaging and filtering of multiple structures to obtain a representative shape using the DAMAVER package (54), as described previously (55). To visualize whether OAS2 forms a monomeric or dimeric conformation in solution, we further processed SAXS data of OAS2 to construct atomistic structural models using the program CORAL (56), as described previously (48). First, we calculated a homology model for OAS2 using a crystal structure of OAS1 (Protein Data Bank, PDB: 4RWN) by means of SWISS-MODEL, using the default web interface (57), and obtained two models corresponding to amino acid residues 6–339 and 345–683. We then combined homology model structural information with merged SAXS data in the program CORAL to obtain the optimal positions and orientations of OAS2 homology models by allowing translation and rotation of the homology models of OAS2. The regions of OAS2 for which no high-resolution data were available were placed as dummy residues by employing a pregenerated library of self-avoiding random loops implemented in CORAL. Using this approach, 20 different conformations were calculated that were then compared using the DAMAVER package (54), which utilizes the program SUPCOMB (58). The goodness of the superimposition of these models was again estimated by the overlap function with normalized spatial discrepancy (NSD). We also performed docking experiments using the CLUSPRO protein-protein docking webserver (59,60). As an input file, we used a monomer atomistic model of OAS2 calculated using CORAL as a receptor and ligand to obtain a set of dimeric atomistic models of OAS2. Note that we did not provide any information on amino acids that could mediate interaction between OAS2 monomers or mediate repulsion (i.e., free docking). We obtained ∼100 atomistic models from CLUSPRO, which were first analyzed using the program CRYSOL (61) and experimentally collated SAXS data. CRYSOL allows identification of atomistic structures by calculating SAXS data from CLUSPRO-derived models and checking their agreement with experimentally collected SAXS data. The CRYSOL selected atomistic structures were aligned to the low-resolution DAMMIN structure using the program DAMAVER.

For OAS1, we obtained two peaks for SEC-SAXS data. Primary data processing in terms of Guinier analysis, dimensionless Kratky analysis, and *P*(*r*) function was performed using the approach outlined for OAS2. To evaluate whether the low-resolution structures obtained from peak 1 of OAS1 data agree with a dimeric structure of OAS1, we designed a dimeric model of OAS1 using the CLUSPRO program (60) and docked high-resolution structure of OAS1 (PDB: 4IG8) to obtain a dimer of OAS1. Similar to OAS2, we performed free docking of OAS1 using CLUSPRO and obtained ∼100 atomistic structures. Using the program CRYSOL (61), we compared the SAXS data for OAS1 with dimeric structures obtained from CLUSPRO (60) to investigate whether the low-resolution structural data agree with the high-resolution models.

### Coimmunoprecipitation assay

For the coimmunoprecipitation assay, HEK293T cells were grown on 100 mm cell culture dishes (Thermo Fisher Scientific) in 10 mL of Dulbecco’s modified Eagle’s medium (DMEM) (Thermo Fisher Scientific) supplemented with 10% fetal bovine serum and grown to 80% confluency. The medium was replaced with fresh DMEM with 10% fetal bovine serum just before transfection. The transfection mixture was made by adding 30 *μ*g of the plasmid to 45 *μ*L (1 g/L) of polyethylenimine (Polysciences, Warrington, PA), and the volume was brought to 5 mL with serum-free DMEM. The transfection mixture was kept at room temperature for 20 min and then added to the cell culture plates dropwise. Recombinant pCDNA3 vector (Thermo Fisher Scientific) containing FLAG-OAS2 and HIS-OAS2 were used to transfect the cells either singly or in combination along with a negative control (empty vector). Cells were incubated at 37°C for 2 days, then harvested using 1 mL of cold phosphate-buffered saline 1× (PBS). Cells were pelleted by centrifugation at 1500 rpm for 5 min at 4°C, resuspended into 1 mL cold phosphate-buffered saline, and repelleted at 2500 rpm for 3 min at 4°C. To acquire the cytoplasmic fractions, the cells were resuspended in 275 *μ*L of cytoplasmic lysis buffer (25 mM HEPES (pH 7.2), 5 mM KCl, 0.5 mM MgCl_2_, 0.5% octylphenoxy poly(ethyleneoxy) ethanol (IGEPAL), and 1× halt protease inhibitor cocktail, EDTA free). The suspension was end-over-end rotated for 5 min, and the insoluble material was pelleted by centrifugation for 5 min at 5000 rpm. The soluble cytoplasmic fraction was retained. The insoluble fraction was further mixed with 275 *μ*L of nuclear lysis buffer (25 mM HEPES (pH 7.2), 350 mM NaCl, 0.01% IGEPAL, 10% w/v sucrose, and 1× halt protease inhibitor cocktail, EDTA free), vortexed, and passed through a 20 gauge needle in triplicate. The suspension was mixed end over end for 10 min and then centrifuged at 4°C for 10 min at 14,000 rpm. Soluble nuclear fractions of the cell lysate were then combined with retained soluble cytoplasmic fractions of the cell lysate. 50 *μ*L of this lysate was set aside for SDS-PAGE gel (pre-IP) and Bradford assay to measure the protein concentration before the IP. The remaining lysate (500 *μ*L) was divided into two 250 *μ*L fractions into microfuge tubes and immunoprecipitation (IP) was performed in total IP buffer (25 mM HEPES (pH 7.2), 175 mM NaCl, 2.5 mM KCl, 0.25 mM MgCl_2_, 0.25% IGEPAL, 5% w/v sucrose, and 1× halt protease inhibitor cocktail, EDTA free). 2.5 *μ*g of anti-HIS, monoclonal anti-FLAG antibody (Sigma-Aldrich, Oakville, ON, Canada) was added to each tube, and 2.5 *μ*g of IgG negative control antibody was added to the lysate where cells were transfected in combination and mixed end over end for 1 h at 4°C. In a 2 mL microfuge tube, 30 *μ*L of protein A/G beads (Thermo Fisher Scientific) was equilibrated in total IP buffer three times for 10 min, then magnetically pelleted to the side wall of the microfuge tube. The buffer was aspirated, and the IP sample was added to the microfuge tube and incubated for 1 h at 4°C with end-over-end mixing. Protein A/G beads were magnetically pelleted, and 50 *μ*L of supernatant was set aside for SDS-PAGE and Bradford assays to measure the protein concentration after IP. The beads were then washed by end-over-end mixing three times for 10 min with 500 *μ*L of total IP buffer, followed by manual pipette mixing. The beads were magnetically pelleted again, the supernatant was aspirated, and the pellet was resuspended in 100 *μ*L of 1× SDS load dye. The solution was heated at 95°C for 5 min, then centrifuged at 14,000 rpm for 5 min at room temperature. 10% SDS-PAGE gels were run at 220 V for 35 min, and Western blots were performed using 50 *μ*g of pre-IP and post-IP and 8 *μ*L of the IP sample. Mouse monoclonal anti-FLAG M2 antibody was purchased from Sigma-Aldrich (Oakville, ON, Canada) (Cat. # F1804), and mouse monoclonal anti-HIS antibodies were purchased from Millipore (Cat. # 05-949).

## Results

### Recombinant human OAS2 adopts a globular structure

**Figure 1:**
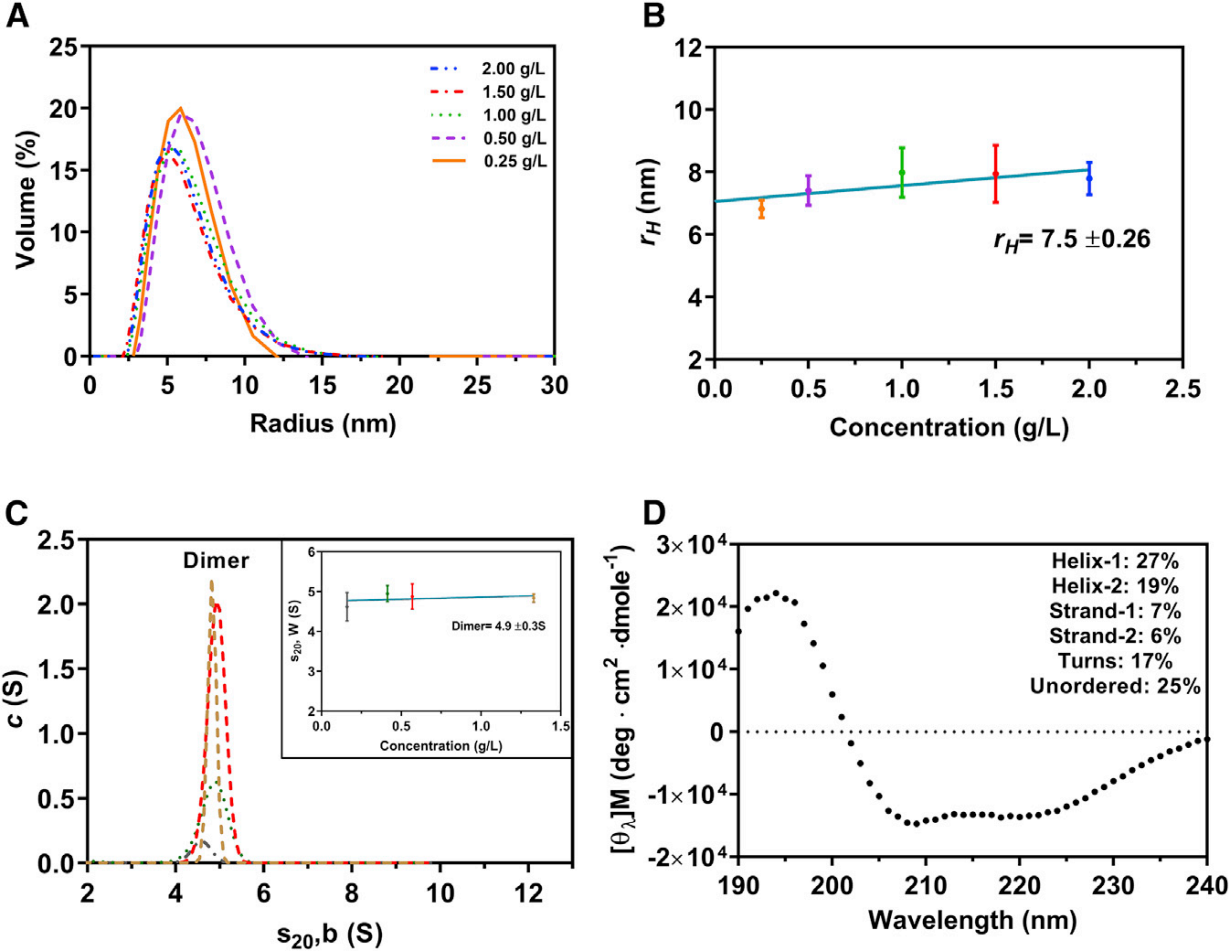
Hydrodynamic properties of recombinant OAS2. (*A*) OAS2 volume distribution versus hydrodynamic radius (*r*_*H*_) as determined by DLS at different protein concentrations (0.25–2 g/L) is shown. (*B*) Concentration dependence of hydrodynamic radius obtained from DLS measurements is shown. The hydrodynamic radius (*r*_*H*_) was determined for OAS2; DLS measurements were taken at protein concentrations in the same range as in (*A*). Error bars represent standard deviation from three replicates. (*C*) Sedimentation velocity (SV) distribution analysis is shown in terms of *c*(_*S*_) at 0.16, 0.41, 0.57, and 1.33 g/L. Inset is the resultant concentration dependence of the SV distribution. The values were corrected to standard conditions (pure water at 20°C), and error bars represent the width of the peak distribution. (*D*) CD spectrum showing the deconvolution of structural features in OAS2 is given. The signal was subtracted from the buffer blank and deconvoluted using DICHROWEB (refer to Materials and Methods). To see this figure in color, go online.

To assess the homogeneity, hydrodynamic properties, and size distribution of OAS2 at different concentrations, we performed DLS and SV-AUC experiments at 0.25–2.0 g/L and 0.16–1.33 g/L, respectively. DLS results suggest multiple components in the distribution which are also reflected by asymmetric peaks from multiple measurements in the OAS2 volume distribution plot (Fig. 1 *A*). Extrapolation of the plot of *r*_*H*_ measured at each concentration to infinite dilution provided a value of 7.5 ± 0.26 nm (Fig. 1 *B*), which is significantly larger than the reported value obtained for OAS1 (3.0 ± 0.3 nm) (34). The plot demonstrated a slight positive slope characteristic of a fast self-associating system. Next, using SV-AUC, we determined the sedimentation coefficient distribution (measured in seconds or Svedberg units S = 10^−13^ s). AUC peaks suggest a contribution from both dimer (dominant) and monomer (minor). We obtained an *s*^0^_20, *w*_ (S) of 4.9 ± 0.3S for OAS2 (Fig. 1 *C*). The *c*(*s*) analysis shows a clear trend of an increase in the signal with increasing OAS2 concentration (see Fig. 1 *C*; Fig. S1) and the presence of more than one species because of a slight shift in *s*-values at the lowest concentration. The frictional ratio is a hydration-dependent parameter that gives an indication of a molecule’s shape in solution. In general, frictional ratios in the range of 1.0–1.3 are observed for spherical molecules, and higher values >1.8 are obtained for elongated macromolecules (62). Based on the AUC data, a frictional ratio of ∼1.0 was calculated (63,64), suggesting a globular shape. In addition, we used the AUC data to calculate the molecular weight of OAS2 (145.8 kDa), which is approximately twice the sequence-based molecular weight (78.78 kDa), suggesting a dimer.

Next, we performed CD experiments on OAS2 to determine the secondary structure content of the protein. The net CD spectrum was deconvoluted using the CDSSTR method and presented features of a folded-globular protein (RMSD value of 0.017). The data suggest that OAS2 consists of helix-1 and 2, constituting 27 and 19%, respectively, of the total protein, and *β*-strand-1 and 2, which form 7 and 6%, respectively, of the total protein. In addition, OAS2 contains 17% turns and 25% unordered structures (Fig. 1 *D*). These values were consistent on analysis with both the CONTIN-LL (RMSD value 0.056) and PYRE^2^ (data not shown) methods.

### OAS2 adopts a dimeric structure in solution

**Figure 2:**
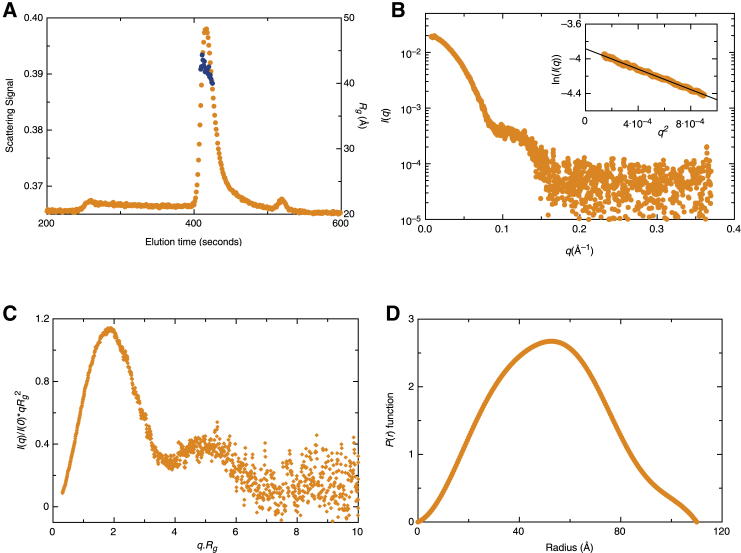
SEC-SAXS analysis of OAS2. (*A*) Signal plot of OAS2 eluting from the in-line SEC connected to SAXS instrumentation is shown. The *y* axis signal is the integral of the ratio of the scattering intensity of the individual frame/the background intensity measured from the buffer. The blue spheres represent the *r*_*G*_-values (*y* axis on *right*) for each frame in the peak. (*B*) OAS2 merged SAXS scattering data obtained from SEC-SAXS experiment are shown. The *x* axis represents momentum transfer (*q*), and the *y* axis (*I*_0_) represents the intensity of the scattered light. Inset to this figure is the Guinier analysis of low-*q* region. (*C*) The dimensionless Kratky plot ((*I*(*q*)/*I*(0) × (*q* × *r*_*G*_)^2^ vs. *q* × *r*_*G*_)) of OAS2 indicates that it has a globular shape in solution. (*D*) The pair-distance distribution function (*P*(*r*)) vs. radius obtained from the GNOM analysis is shown. To see this figure in color, go online.

To obtain solution structures of OAS2, we collected data using a coupled SEC-SAXS setup in which SAXS data were collected every 3 s during elution from a size-exclusion column. The sample eluted as a predominant peak at ∼400 s (Fig. 2 *A*). The signal in the chromatogram is the integral of the ratio of the scattering intensity of the individual frame/the background intensity measured from the buffer. Individual scattering intensity profiles that provided uniform *r*_*G*_ distribution and displayed monodispersed distribution were merged using the program PRIMUS (47) (Fig. 2 *B).* Guinier analysis of the merged data demonstrated a monodispersed preparation (*inset* to Fig. 2 *B*). The merged data from Fig. 2 *B* were also used to perform dimensionless Kratky analysis (plot of (*I*(*q*)/*I*(0)) × (*q* × *r*_*G*_)^2^ vs. *q* × *r*_*G*_) that is particularly useful in determining global protein folding. The plot presented in Fig. 2 *C* suggests a Gaussian distribution and a well-defined maximum, confirming that OAS2 is a well-folded protein. For a globular-shaped protein in solution, a dimensionless Kratky plot displays a well-defined maximal value of 1.1 at *q* × *r*_*G*_ = $\sqrt{3}$. As presented in Fig. 2 *C*, OAS2 does not adopt an extended conformation in solution.

**Figure 3:**
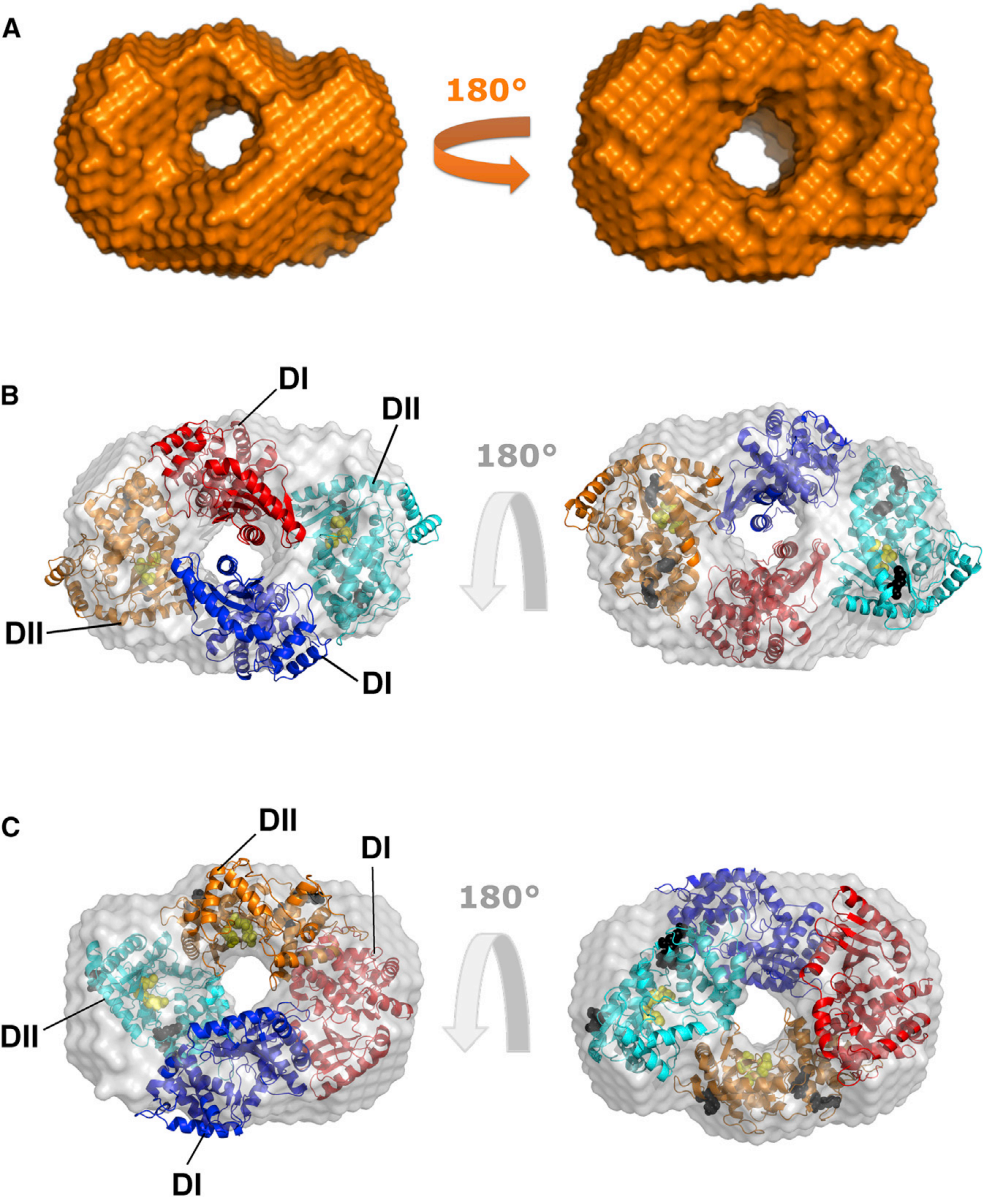
OAS2 ab initio modeling. (*A*) The DAMMIN ab initio model for OAS2 is given, showing protein surface representation and rotation at 180°. (*B*) Superimposition of the atomistic structure of OAS2 calculated using the program CORAL on the averaged-filtered ab initio low-resolution structure obtained from DAMMIN and DAMFILT is shown. (*C*) Superimposition of the atomistic structure of OAS2 calculated using the program CLUSPRO on the averaged-filtered ab initio low-resolution structure obtained from DAMMIN and DAMFILT is shown. In (*B*) and (*C*), the dsRNA binding sites are represented by black spheres, whereas the aspartic acid residues are indicated by yellow spheres. To see this figure in color, go online.

We calculated the radius of gyration (*r*_*G*_) and forward angle scattering (*I*_0_) using the Guinier approximation (65) (Table 1). The *P*(*r*) analysis (which represents a histogram of the interelectron distances within the structure) (66,67) was performed using the GNOM software (67). This analysis yielded a roughly bell-shaped distribution for OAS2, suggesting a globular (as opposed to extended) conformation in solution (Fig. 2 *D*), in agreement with the frictional ratio obtained from SV-AUC experiments. Globular proteins usually have a bell-shaped *P*(*r*) curve, whereas elongated proteins have an extended tail (68). Based on the *P*(*r*) analysis, we obtained the *r*_*G*_ and *D*_*max*_ of OAS2 as 42 ± 0.25 Å and 110 Å, respectively (Table 1). In addition to *D*_*max*_, the ratio of *r*_*G*_/*r*_*H*_ can also provide an indication about the solution conformation of macromolecules. In this study, we obtained an *r*_*G*_/*r*_*H*_ ratio of 0.56, which also suggests a globular conformation of OAS2. r_*G*_/r_*H*_ of approximately of 0.7 has been reported for globular proteins and greater than 2.0 for elongated rod-like structures (35,69,70). A total of 13 low-resolution structures for OAS2 were calculated using the program DAMMIN, with *χ*^2^-values of ∼0.77, suggesting a reliable agreement between the experimentally collected SAXS data and data obtained from each low-resolution structure (52) (see Fig. S3). Subsequently, the structures were aligned and averaged, and a representative structure was calculated using the program DAMAVER (54). The NSD of 0.55 ± 0.02 suggests that all 13 low-resolution structures are highly similar to each other (52). Overall, the SAXS analysis suggests that the OAS2 adopts a well-defined donut-shaped structure with a pronounced central cavity (Fig. 3 *A*). Consistent with dimeric OAS2, a volume-based molecular weight of ∼165 kDa was obtained from the solution structure, nearly twice the mass of the sequence-derived molecular weight (78.78 kDa) of OAS2.

To obtain structural insights into dimeric OAS2, we first designed a homology model of OAS2 using an OAS1 crystal structure (71), and the SWISS-MODEL software (57). Next, we employed the rigid-body modeling program CORAL (56) to model the OAS2 structure using SAXS data and the homology model of OAS2 as input parameters, as described previously (48). Because the hydrodynamic analysis suggested that OAS2 predominantly exists as a dimer in solution, we calculated 20 models using P2 symmetry, which yielded *χ*^2^-values of ∼0.6 (see Fig. S3). Although the low *χ*^2^-values are indicative of an optimal agreement between the SAXS data for OAS2 and structural models derived using the CORAL software, we obtained NSD values of 2.4 ± 0.11, suggesting that multiple OAS2 dimer conformations are possible. Subsequently, we also investigated whether the structures calculated using the CORAL program agree with the low-resolution structure obtained from DAMMIN. We found that indeed, the vast majority of the CORAL-derived structures contained a central cavity around which four individual OAS subunits could be accommodated (two from each OAS2 monomer). A representative example is presented in Fig. 3 *B*. The atomistic models derived using CORAL suggested that although OAS2 forms a dimer in solution, the catalytically active domains (DII) are located across the central cavity as opposed to being side by side. In parallel, we utilized CLUSPRO (60) to perform docking experiments and obtained a number of models indicating that the OAS2 dimer can additionally include a side-by-side assembly of the catalytically active domains. A representative example is presented in Fig. 3 *C*, for which a *χ*^2^-value of 1 was obtained (see Fig. S3). Therefore, based on the SAXS envelopes obtained, the orientation of the two OAS2 protomers in the context of a dimer cannot be unambiguously defined.

### OAS2 self-association is observed in a cellular context

**Figure 4:**
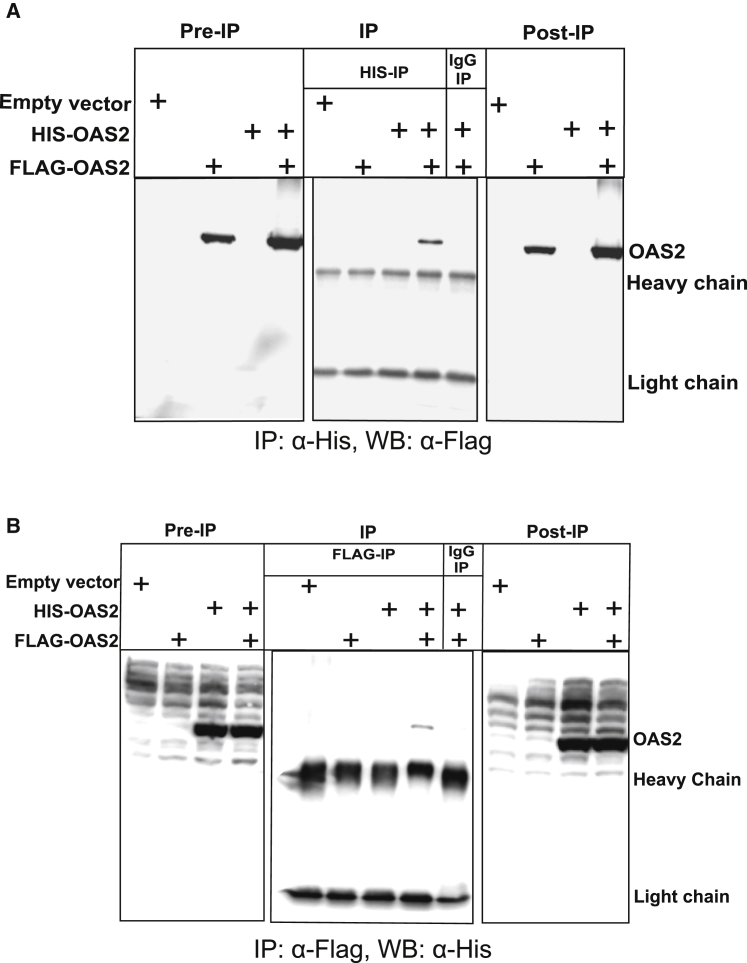
Immunoprecipitation of OAS2 in cells. (*A*) Immunoprecipitation of OAS2 using anti-His antibody is shown. HEK293T cells were transfected with empty vector, FLAG-OAS2, HIS-OAS2, and in combination with both. Cells were lysed and protein immunoprecipitated using anti-His antibodies and IgG negative control. Immunoprecipitated (IP) protein was loaded on the 10% SDS-PAGE gel, and equal amounts of protein were loaded before (pre-IP) and after (post-IP). The Western blot was performed using anti-FLAG antibodies. (*B*) Immunoprecipitation of OAS2 using FLAG antibodies. The same immunoprecipitation procedure was followed as in (*A*), except anti-FLAG antibodies were used for immunoprecipitation and the Western blot was performed using anti-His antibodies.

To confirm OAS2 self-association in the context of a cellular background, we transfected HEK293T cells with combinations of empty pCDNA3 vector, FLAG-tagged OAS2, and HIS-tagged OAS2 and performed coimmunoprecipitation experiments. Fig. 4 *A* shows the results as detected by Western blot using anti-FLAG for visualization. Before immunoprecipitation with anti-HIS (pre-IP, *left panel*), FLAG-OAS2 can be efficiently detected. FLAG-OAS2 can also be detected after immunoprecipitation with anti-HIS (IP, *middle panel*) only under conditions in which both FLAG-OAS and HIS-OAS2 are co-transfected into cells. This suggests self-association between at least two OAS2 protomers. Under all other control immunoprecipitation conditions examined (including immunoprecipitation with mouse IgG, negative control), no FLAG-OAS2 was detected. The reciprocal experiments using anti-FLAG immunoprecipitation with anti-HIS detection by Western blot demonstrated similar results, again suggesting OAS2 self-association (Fig. 4 *B*).

### OAS2 dimerization is independent of CAFAKA mutation

**Figure 5:**
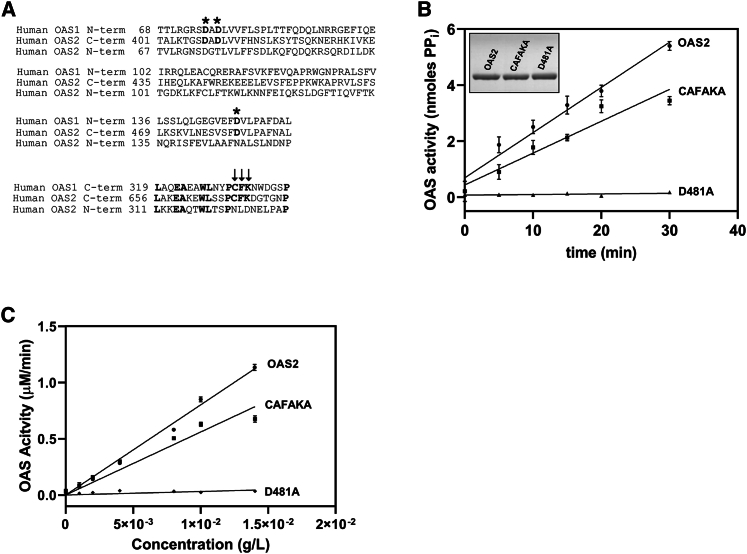
Characterization of OAS2 mutants. (*A*) MUSCLE sequence alignment is shown. The sequence homology of OAS enzymes near the carboxyl terminal shows the conserved CFK residues represented by arrows, and the conserved aspartic acid residues are indicated by asterisks. (*B*) OAS2 activation comparison is shown. Activation assay (refer to Materials and Methods) comparing the activity (nmoles of PP_i_) of OAS2 and mutants D481A and CAFAKA is shown. SDS-PAGE analysis of purified OAS2, D481A, and CAFAKA is shown (*inset*). (*C*) OAS2 activation assay at varying concentrations (0.0–0.0143 g/L) is shown, comparing the activity (*μ*M/min) of OAS2 and D481A and CAFAKA mutants. Error bars represent standard deviation from three replicates.

To further probe the importance of dimerization in OAS2 activity, we expressed and purified OAS2 mutants from HEK293T cells. Previous studies indicated that mutation of three C-terminal amino acids in the catalytic domain of OAS1 or OAS2 (CAFAKA mutant) causes a complete loss of activity in OAS1 and OAS2 purified from bacterial and insect cells, respectively, potentially because of the prevention of OAS self-association (14,31). Mutation of an active-site aspartic acid residue (D481A) prevents ATP binding and therefore activity (14,31). The sequence alignment of human OAS1 and OAS2 is shown in Fig. 5 *A*, highlighting the three active-site aspartic acid residues with asterisks and the C-terminus CFK residues with arrows. OAS2, CAFAKA, and D481A were expressed and purified simultaneously (Fig. 5 *B*, *inset*). To determine whether the CAFAKA mutant impacts enzymatic activity, we used an established OAS activity assay (28,72) that detects the production of pyrophosphate byproduct, which forms during 2-5A synthesis. As expected, OAS2 was enzymatically active, and D481A did not produce 2-5A above baseline. However, the CAFAKA OAS2 mutant displayed enzymatic activity, albeit to a lesser extent compared with OAS2 (Fig. 5 *B*). CD analysis of D481A suggests a well-folded protein consistent with wild-type features (data not shown). We then repeated the assay at a single time point over a range of protein concentrations (0–1.43 × 10^−2^ g/L) to assess the impact of low concentrations that may disrupt dimer formation on activity. As expected, D481A did not display any enzymatic activity, but OAS2 and the CAFAKA mutant were active even at low protein concentrations, suggesting that these residues are not sufficient for dimerization (Fig. 5 *C*).

### Hydrodynamic studies of OAS1

**Figure 6:**
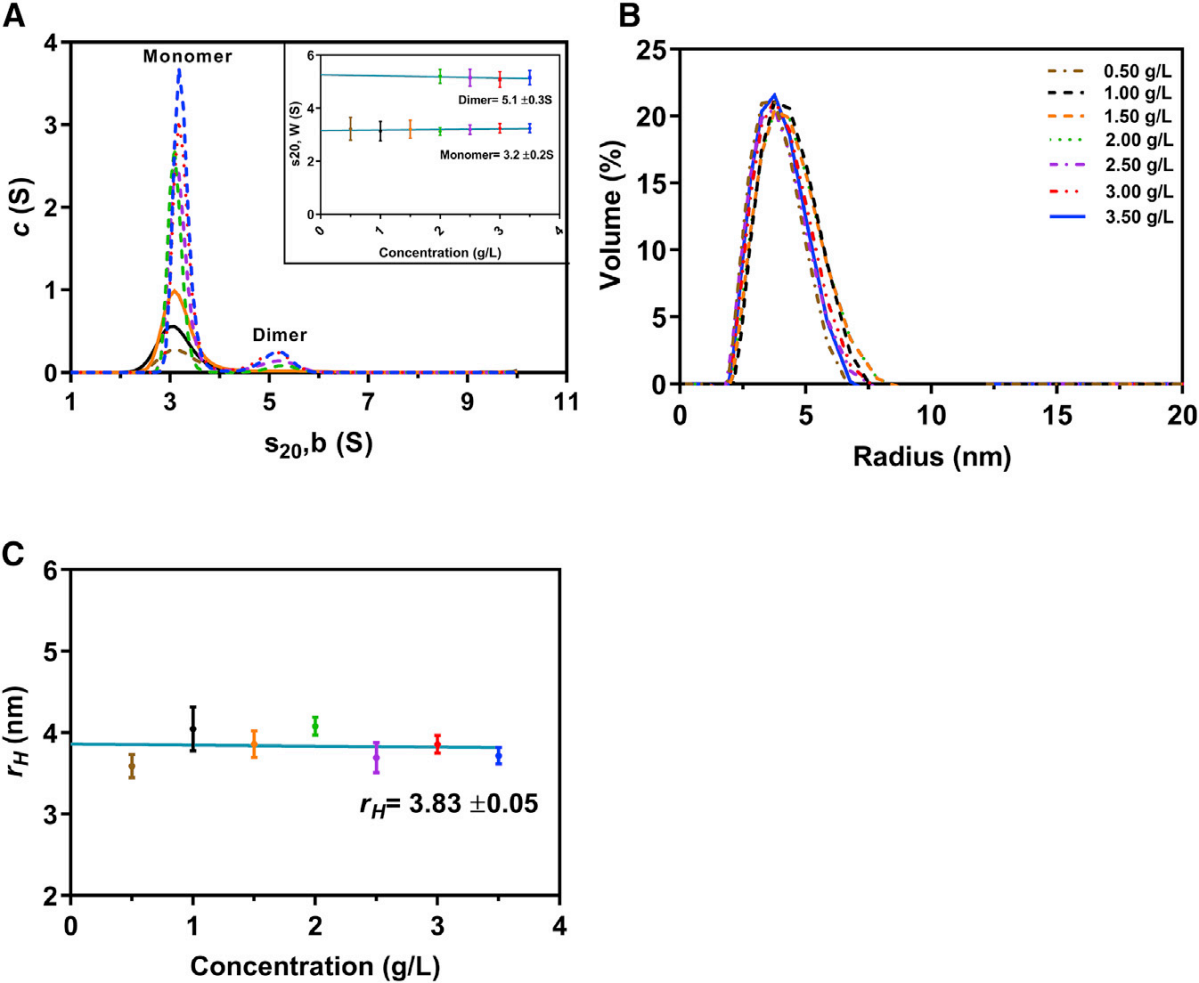
Hydrodynamic properties of recombinant OAS1. (*A*) SV distribution analysis in terms of *c*(_*S*_) at 0.5–3.5 g/L is shown. Inset is the resultant concentration dependence of the SV distribution. The values were corrected to standard conditions (pure water at 20°C). Error bars represent the width of the peak distribution. (*B*) Percent volume distribution profile is shown. OAS1 volume distribution by DLS in percent versus the hydrodynamic radius (*r*_*H*_) at different protein concentrations (0.5–3.5 g/L) is shown. (*C*) Concentration dependence of the hydrodynamic radius was obtained from DLS measurements. The hydrodynamic radius (*r*_*H*_) was determined for OAS1 by DLS at protein concentrations in the range as in (*B*). Error bars represent standard deviation from three replicates. To see this figure in color, go online.

Our previous studies on OAS1 using SV-AUC at low concentrations (up to 0.8 g/L) suggested that OAS1 is monomeric in solution (34). In this study, we investigated the OAS1 size distribution over a much broader range of concentrations (0.5–3.5 g/L) to investigate whether OAS1 forms dimers in solution using the SV-AUC and DLS methods. The *c*(*s*) analysis showed an increase in signal intensity with increases in OAS1 concentration (see Fig. S2) and yielded a single peak for OAS1 from 0.5 to 1.5 g/L, with the *s*^0^_20, *w*_ of 3.2 ± 0.2 S, which is consistent with the previously published values (34). However, our SV experiments for OAS1 at higher concentrations (2.0–3.5 g/L) provided an additional peak at 5.1 ± 0.3 S, suggesting that above the concentration range of ∼2 g/L, OAS1 could exist as a dimer, albeit at smaller proportion to the monomer (Fig. 6 *A*). We also calculated the molecular mass of both OAS1 monomer and dimer using *SEDFIT*, which yielded ∼44.9 and 91.6 kDa, respectively; a single frictional ratio of 1.4 in continuous *c*(*s*) distribution model was used, and monomer and dimer distribution curves were integrated into SEDFIT to determine the molar mass (see Fig. S4). DLS in the same concentration range (0.5–3.5 g/L) shows the average volume-weighted *R*_*h*_ distribution (Fig. 6 *B*). The hydrodynamic radius (*r*_*H*_) extrapolated to infinite dilution was 3.83 ± 0.05 nm, a value consistent with a distribution containing mainly monomers (Fig. 6 *C*).

### Solution structure determination of OAS1

Previously, we published a low-resolution structure of OAS1 (34) based on the SAXS data collected using our in-house SAXS device. Although we followed rigorous quality control checks to ensure that samples were monodispersed, the in-house instruments lacked the capacity of separating low concentrations of higher-molecular-weight material. In light of the AUC data that suggested OAS1 could potentially form a dimer in solution at higher concentrations, we sought to further investigate this observation using a synchrotron source equipped with an SEC-SAXS setup allowing separation of OAS1 species. Furthermore, the synchrotron radiation offers better usable data at higher angles (2*θ*) than the home source.

**Figure 7:**
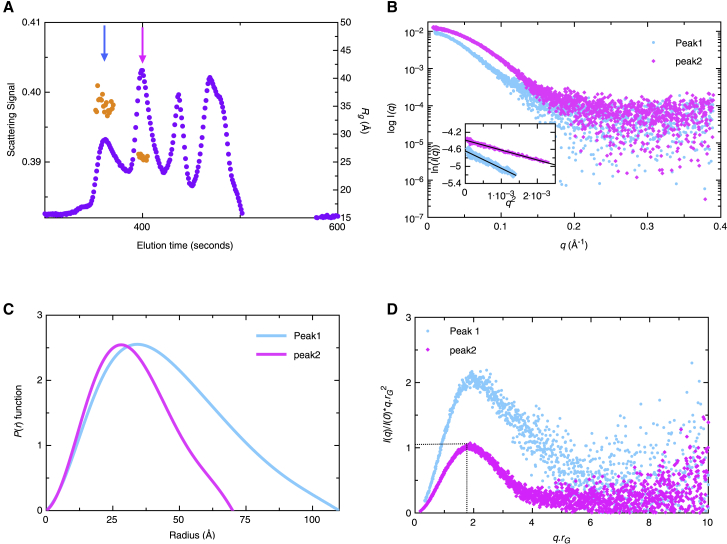
Synchrotron SEC-SAXS data for OAS1. (*A*) Signal plot of the OAS1 eluting from in-line SEC is given. The signal is the integral of the ratio of the scattering intensity of the individual frame/the background intensity measured from the buffer. Peak-1 (*blue arrow*) and peak-2 (*pink arrow*) show a dimer and monomer, respectively. The orange spheres represent the *r*_*G*_-values (*y* axis on *right*) for each frame in the peak. (*B*) OAS1 merged SAXS scattering data (dimer (in *blue*) and monomer (*pink*)) are shown. The *x* axis represents momentum transfer (*q*), and the *y* axis (*I*_0_) represents the intensity of the scattered light. The inset to this figure is the Guinier analysis of the low-*q* region. (*C*) *P*(*r*) plot for OAS1 is shown. The pair distribution function versus particle radius obtained from the GNOM analysis is shown. (*D*) Dimensionless Kratky plots ((*I*(*q*)/*I*(0) × (*q* × *r*_*G*_)^2^ vs. *q* × *r*_*G*_)) of OAS1 dimeric (*blue*) and monomeric (*pink*) fractions are given, indicating that the monomeric fraction adapts a globular shape in solution. To see this figure in color, go online.

The SEC-separated fractions were subjected to x-ray radiation, which provided a scattering profile as presented in Fig. 7 *A*. For OAS1, we obtained four peaks at different time points (Fig. 7 *A*). Data processing of the first (peak-1, *blue arrow*) and the second (peak-2, *pink arrow*) peak provided sufficient scattering signal such that we could obtain the *r*_*G*_ and *D*_*max*_ from both peaks. The raw scattering data obtained from peak-1 (*blue*) and peak-2 (*pink*) show different scattering patterns, suggesting that a unique envelope can be extracted from each (Fig. 7 *B*). We also performed Guinier analysis (*inset* to Fig. 7 *B*) for both peaks, and the linearity observed by fitting the low-*q* data suggests that both peaks are monodispersed. Subsequently, we performed the *P*(*r*) analysis to obtain the *r*_*G*_ and *D*_*max*_ values from both peaks. The *P*(*r*) analysis for peak-2 resembled the previously published data (Fig. 7 *C*), including the *r*_*G*_ (24.88 ± 0.02 Å) and *D*_*max*_ (70.0 Å), indicating that peak-2 corresponds to a monomeric version of OAS1. Furthermore, similar to OAS2, we performed a dimensionless Kratky analysis. As presented in Fig. 7 *D*, this analysis suggests that peak-2 of OAS1 adapts globular conformation in solution, whereas peak-1 does not. Because peak-1 represents a higher molecular weight than peak-2, we investigated whether it potentially represents a dimeric version of OAS1. The *P*(*r*) analysis for peak-1 is consistent with an extended tail, suggesting the shape should be more cylindrical or ellipsoidal (Fig. 7 *C*). The *R*_*g*_ obtained from *P*(*r*) plots for peak-1 and peak-2 were 34.10 ± 0.10 Å and 24.88 ± 0.02 Å, respectively. The *D*_*max*_ determined for peak-1 and peak-2 are 110 and 70 Å, respectively (Table 2).

**Table 2:** Experimental and Predicted Hydrodynamic Parameters for Recombinant OAS1

	Peak-1	Peak-2
**Guinier Data**		

*r*_*G*_ (Å)	34.10 ± 0.10	24.88 ± 0.02
*I*_0_	0.0058 ± 1.7 × 10^5^	0.014 ± 4.2 × 10^5^
*q* × *r*_*G*_ range	0.29–1.30	0.31–1.28
Data points	6–133	28–195

**Real-space data**		

*r*_*G*_ (Å)	35.66 ± 0.10	24.62 ± 0.04
*D*_*max*_ (Å)	110	70
*I*_0_	0.0057 ± 1.9 × 10^5^	0.013 ± 2.6 × 10^5^
*q* range (Å^−1^)	0.009–0.346	0.017–0.30
Sequence *M*_*w*_ (kDa)	41.74	41.74
*M*_*w*_ calculated (kDa) SAXS MoW	78, dimer	39, monomer
s_20, w_ (S)	5.1 ± 0.3	3.2 ± 0.2
*r*_*H*_ (nm)	N/A	3.83 ± 0.05
*χ*^2^ DAMMIN	∼0.70	∼1.40
NSD DAMMIN	1.0 ± 0.05	0.71 ± 0.08

**Figure 8:**
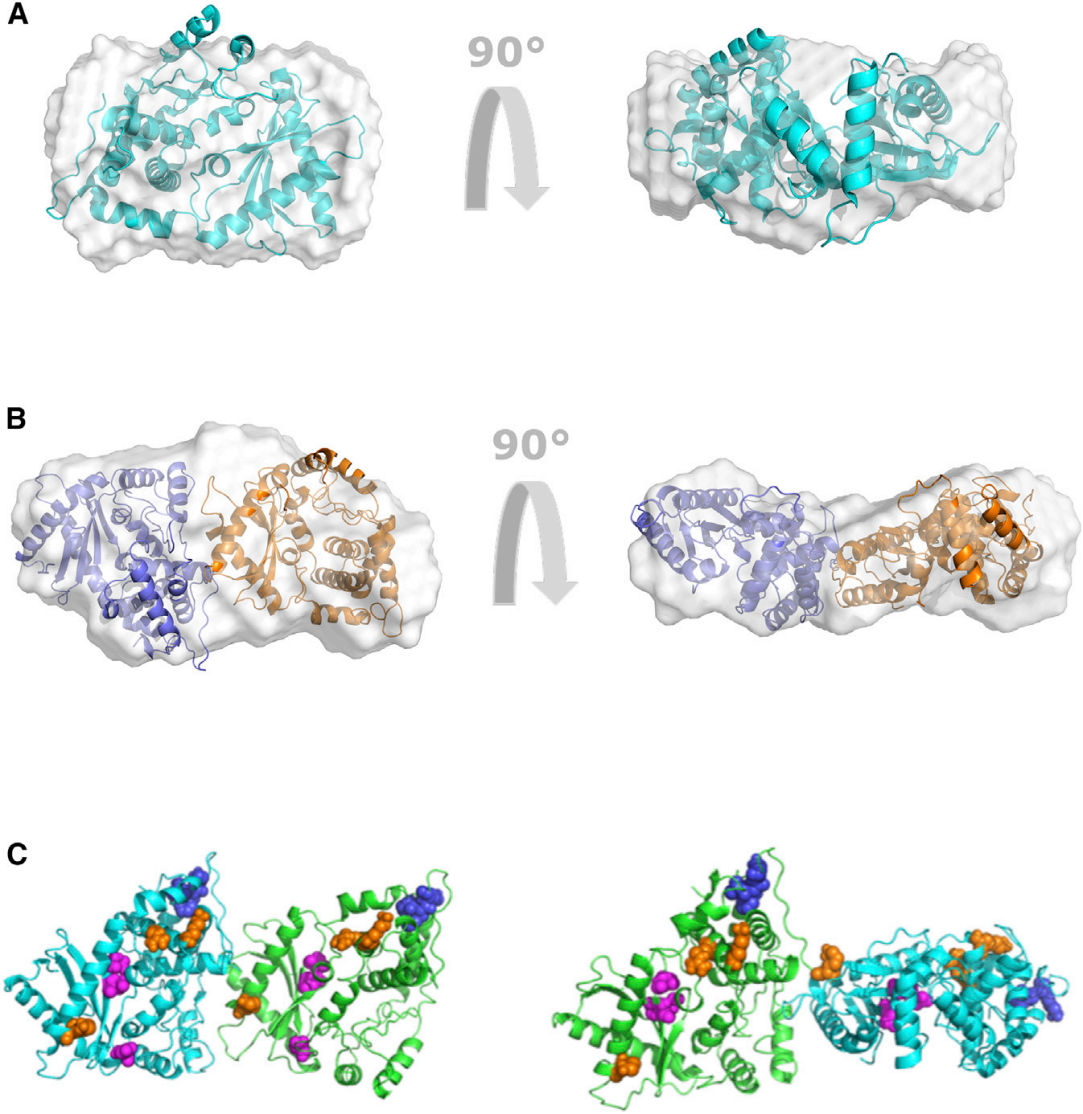
OAS1 ab initio modeling. (*A*) DAMMIF models of OAS1 are shown. Models were generated from the scattering data using DAMMIF. The high-resolution crystal structure of OAS1 (PDB: 4IG8) was superimposed on the models. (*B*) DAMMIN models of OAS1 dimer are shown. Models were generated from the scattering data of peak-1 and fitted to the rigid-body modeling of OAS1 dimer using CLUSPRO. (*C*) OAS1-OAS1 were docked using the program CLUSPRO, and two predominant OAS1 dimer confirmations were chosen using the program CRYSOL. dsRNA binding sites (*orange*), aspartic acid residues (*magenta*), and CFK residues (*blue*) are shown in the dimeric OAS1 molecule. To see this figure in color, go online.

We calculated 13–20 structural models for both peak-1 and peak-2, which were then rotated, aligned, and filtered as above to obtain a representative shape. We obtained the *χ*-values of ∼0.7 and ∼1.4 for peaks 1 and 2, respectively, which represent an agreement between experimentally collected SAXS data and data backcalculated from the low-resolution structures (see Fig. S3). The NSD values of 1.0 ± 0.05 for peak-1 suggest that the low-resolution structures were highly similar to each other. Similarly, we obtained an NSD value of 0.71 ± 0.08 for structures calculated using data from peak-2. The low-resolution structure generated for peak-2 (Fig. 8 *A*) is similar to the previously published model obtained from the in-house scattering data (34). The structure calculated from peak-1 data is more elongated (Fig. 8 *B*), further indicating that peak-1 could represent a dimeric conformation. To determine whether the OAS1 structure obtained from peak-1 data can accommodate two OAS1 monomers, we performed docking using CLUSPRO (60). Next, we used the CRYSOL (61) program to identify the potential conformations from a population of CLUSPRO-docked OAS1 dimer structures, using the peak-1 SAXS data as a restraint. This analysis suggested that OAS1 could adopt a dimer in two conformations (Fig. 8 *C*; Fig. S3). The *χ*-value describing an agreement between the experimentally collected SAXS data and data calculated from CLUSPRO models for both clusters were 0.46 and 0.47, suggesting that both dimeric conformations are equally likely (Fig. 8 *C*). Interestingly, CFK residues (Cys668, Phe669, and Lys670), active-site aspartic acid residues (Asp408, Asp410, and Asp481), and the RNA binding interface are all surface exposed (Fig. 8 *C*).

## Discussion

Although strong structural data on OAS1 are available, the structural features of OAS2 and the mechanism through which OAS2 produces 2-5A chains are not fully understood. Furthermore, it is still not clear whether 2-5A chains produced by OAS2 require multiple OAS active sites within the OAS2 dimer. We had previously purified OAS2 to homogeneity and preliminarily demonstrated a dimer (28). In the absence of any structural and hydrodynamic information on OAS2, we used a combination of different biophysical techniques, including AUC, DLS, CD, and SAXS, to investigate the solution properties and hydrodynamic parameters of OAS2. SV experiments and DLS suggest that OAS2 is in a monomer-dimer equilibrium that heavily favors dimer in solution. By SEC-SAXS, we demonstrated that OAS2 molecules are dimerized with a defined central cavity in the middle of the structure sufficiently large for nucleotide and 2-5A entry and exit. Based on low-resolution structures, we hypothesize that both domains I and II of OAS2 are involved in the interaction with domains I and II of another OAS2 protomer. It is evident that the OAS2 atomistic models derived using CORAL and CLUSPRO are suggesting different arrangements of monomers; however, both methods indicate that OAS2 is dimeric (Fig. 3, *B* and *C*). The differences in the arrangement of monomeric units are because both methods employ different algorithms and restraints (e.g., CORAL uses SAXS data and simulated annealing protocols, whereas CLUSPRO does not). Therefore, rather than presenting the results from one of the two methods, we are presenting data from both methods to demonstrate that both conformations are possible and that without experimental evidence of the high-resolution structure of OAS2 dimer bound to RNA, it is very difficult to select any of the conformations with confidence. Coimmunoprecipitation assays suggest that OAS2 does indeed self-associate in a cellular background, as previously suggested (14).

The specific contributions of the individual OAS2 domains are largely unknown in the context of the dimer, although both domains are required for catalytic activity (21,28). Weak binding of DI in isolation to poly I:C-bound Sepharose beads was observed, suggesting that this domain has lost the ability to bind dsRNA (21). This observation is supported by the absence of a stretch of 54 amino acid residues (residues 104 and 158) that have been previously identified as part of the dsRNA binding site for the murine isoform of OAS1 (73). This region does not show striking homology within the corresponding residues of OAS2, except for the last amino acids (FDVLPAF), which are identical in DII but only partially conserved (FEVLAAF) in DI, suggesting loss of dsRNA binding ability in OAS2 DI (21). These observations are further supported by computational models of DI and DII of OAS2 docked to dsRNA. Only DII, but not DI, could be docked to dsRNA with favorable energetics by computational approaches (74, 75, 76), supporting the hypothesis that DI has lost its ability to bind dsRNA. Three important, nondispensable dsRNA binding residues conserved in hOAS1 are also conserved in DII, but not in DI, of OAS2 (27), suggesting the DI is potentially unable to bind dsRNA.

**Figure 9:**
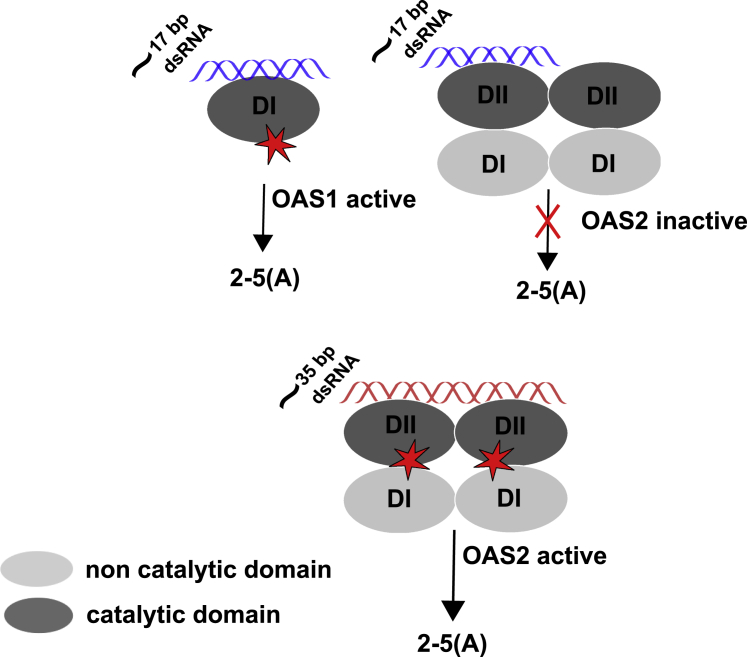
Model of OAS2-dsRNA interaction. (*A*) OAS1 recognizes ∼17 bp dsRNA to be activated, and longer dsRNAs are identified indiscriminately. However, OAS2 is only active as a dimer and requires ∼35 bp dsRNA to interact with both catalytically active domains that potentially cause a confirmation change in the dimeric structure catalyzing the 2-5A chains. Short dsRNAs that interact with either of the two active domains are insufficient to activate the full-length dimeric protein. To see this figure in color, go online.

It has been previously suggested that the minimal dsRNA length requirement for OAS1 activation is 17 bp, and the protein-dsRNA interface involves two minor grooves (13). We have recently shown that a minimum of ∼35 bp dsRNA is required for OAS2 activity (28). Because OAS2 consists of two OAS-like domains (DI and DII), dsRNA interactions with OAS2 would require the interaction of both OAS2 domains with four minor grooves that set the dsRNA length requirement at ∼35 bp (28). However, based on the above information that OAS2 DI may have lost its ability to bind dsRNA coupled with the low-resolution OAS2 structural data obtained from SEC-SAXS, we propose a model of dsRNA-OAS2 interaction that supports a longer dsRNA requirement than OAS1 (Fig. 9). Because SAXS is a low-resolution technique and computational modeling is dependent on a number of parameters, overall, this analysis only suggests that OAS2 forms a dimer in solution, accommodating four OAS core domains tightly bound to each other (Fig. 3, *B* and *C*). Incorporating previous biochemical observations, we propose the DI of one OAS2 protomer is tightly bound to the DI of another OAS2 protomer. Similarly, DII is also bound to DII of another OAS2 protomer providing it an overall globular shape. We hypothesize that dsRNA interacts with both catalytically active domains of the OAS2 dimer and requires interaction with a minimum of four minor grooves of dsRNA provided by ∼35 bp dsRNA. In addition, we also suggest that OAS2 protomers have very high affinity for each other, as is shown by the immunoprecipitation assays and supported by a tight dissociation constant of 5.1 nM previously observed (14). Monomeric OAS2 is shown to be catalytically inactive, and only the dimeric protein is catalytically active (14,28). However, caution should be taken because the SAXS data presented do not rule out alternative orientations of the individual protomers in the dimer and await the structure of OAS2 bound to dsRNA.

The residues C668, F669, and K670 present on the C-terminal end of OAS2 have been previously suggested to be important for its activity and dimerization (14). We made CAFAKA and D481A mutants; CAFAKA was not well expressed (∼12-fold reduction compared to wild-type OAS2). D481A, as expected, did not show any activity, as shown previously (14), but surprisingly, CAFAKA retained significant 2-5A catalysis, albeit modestly lower than the wild-type OAS2. We confirmed this activity at low concentrations (0–1.43 × 10^−2^ g/L). This result is in contrast with Sarkar et al., who suggested that CAFAKA leads to complete loss of activity (14). However, our mutants were expressed in HEK293T cells, and that of Sarkar et al. were expressed in TNT rabbit reticulocyte lysates.

Although our results suggest OAS2 adopts a dimer conformation under a wide range of protein concentrations, we observed that OAS1 adopts a dimer only at higher concentrations well above those that would be observed physiologically and is a minor species relative to the monomer. However, there are a limited number of reports suggesting that OAS1 self-association may be mechanistically important. Wang et al. recently suggested mechanistic differences in OAS1 activation with longer dsRNAs, and their results questioned whether the crystal structure of OAS1 in complex with 18 bp dsRNA is representative of a fully activated form of OAS1 (13,77). Although multimerization in OAS1 was not shown directly, it was suggested that strong activation of both OAS1 and OAS3 by poly I:C and long dsRNA cannot be explained by a simple interaction of a monomeric OAS enzyme with the long dsRNA (77). OAS enzymes belong to nucleotidyltransferase superfamily, which includes cyclic GMP-AMP synthase-like (cGAS) that also undergoes self-association (78,79) to mediate antimicrobial immunity through an interaction with dsDNA (80). Both OAS and cGAS share a similar active site organization and have a common structural fold (78,81). dsRNA or dsDNA binding of OAS or cGAS is followed by a conformational change in the active site (13,82). OAS and cGAS consist of three conserved aspartic acid catalytic residues and demonstrate length-dependent activation (28,77,80). cGAS produces cyclic 2′5′ cGAMP instead of linear 2′5′ A in OAS (78,80). The optimal dsDNA required for cGAS activity was shown to be several kilobases as compared with the 22–25 bp bound to monomeric cGAS (80). It was later shown that cGAS multimerization is key for cGAS activation by long dsDNA (83). Although speculative at this point, we suggest OAS1 self-association is relatively low affinity and that dsRNA binding (also relatively low affinity for an RNA-protein interaction) may enhance OAS1 self-association, which may explain the differences seen in OAS1 activity by minimal OAS activators and long dsRNA activators and/or poly I:C.

In conclusion, we have demonstrated the hydrodynamic parameters of OAS2 and obtained the first, to our knowledge, low-resolution structures of OAS2 dimer. In addition, a combination of biophysical tools has provided us structural information about in-solution structures, stability, and homogeneity of OAS enzymes. This information could potentially help us to better understand how OAS enzymes exist in cells and interact with other proteins and nucleic acids and provides us with a better understanding of the OAS role during viral infections.

## Supporting Material

Document S1. Figs. S1–S4

Document S2. Article plus Supporting Material
