# Structural and Hydrodynamic Characterization of Dimeric Human Oligoadenylate Synthetase 2

**DOI:** 10.1016/j.bpj.2025.02.025

**Published:** 2025-03-13

**Authors:** Amit Koul, Danielle Gemmill, Nikhat Lubna, Markus Meier, Natalie Krahn, Evan P. Booy, Jörg Stetefeld, Trushar R. Patel, Sean A. McKenna

## Main text

(Biophysical Journal *118*, 2726–2740; June 2, 2020)

The authors identified a minor correction to Fig. 1 *C*. Further studies of this system in the authors’ group identified that the method used to analyze the AUC data was not appropriate given the data quality and the presence of a reducing agent; as a result, the determined sedimentation coefficient of 4.9S was an underestimate and was inconsistent with the rest of the experimental results, which strongly support OAS2 dimer formation. We therefore sought external advice and reanalyzed the OAS2 data by using the robust US3 program, which includes multiple optimization steps capable of handling the data acquired. US3 is useful in both single- and multi-component systems; can determine a sedimentation coefficient distribution of the sedimenting proteins; and provides accurate values for molecular weight, frictional ratio, and sedimentation coefficient for proteins. The newly reported value of 6.5S is consistent with an OAS2 dimer.

The corrected version of Fig. 1 *C* and a corrected legend appear below.

**Figure:**
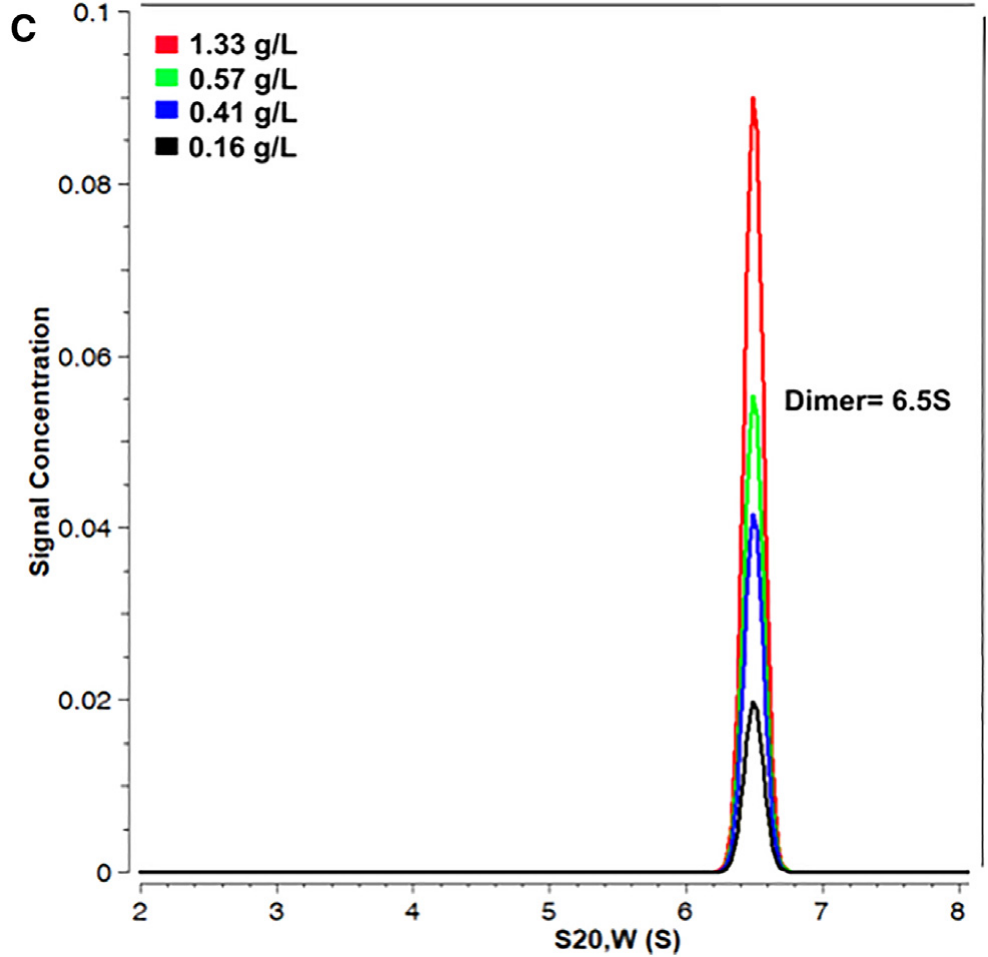
Figure 1C. Hydrodynamic properties of recombinant OAS2. **(***C***)** Sedimentation velocity (SV) distribution analysis in terms of *c(*_*S*_*)* at 0.16, 0.41, 0.57, and 1.33 g/L.

