# Supplementary material for: Structural and Hydrodynamic Characterization of Dimeric Human Oligoadenylate Synthetase 2
Source: Biophys J. 2020 May 1;118(11):2726–40. doi: 10.1016/j.bpj.2020.04.025 (PMC7264852; doi:10.1016/j.bpj.2020.04.025)
Supplement: Document S1. Figs. S1–S4 [file mmc1.pdf]

Biophysical Journal, Volume *118*

Supplemental Information

Structural and Hydrodynamic Characterization of Dimeric Human Oligoadenylate Synthetase 2

Amit Koul, Darren Gemmill, Nikhat Lubna, Markus Meier, Natalie Krahn, Evan P. Booy, Jörg Stetefeld, Trushar R. Patel, and Sean A. McKenna

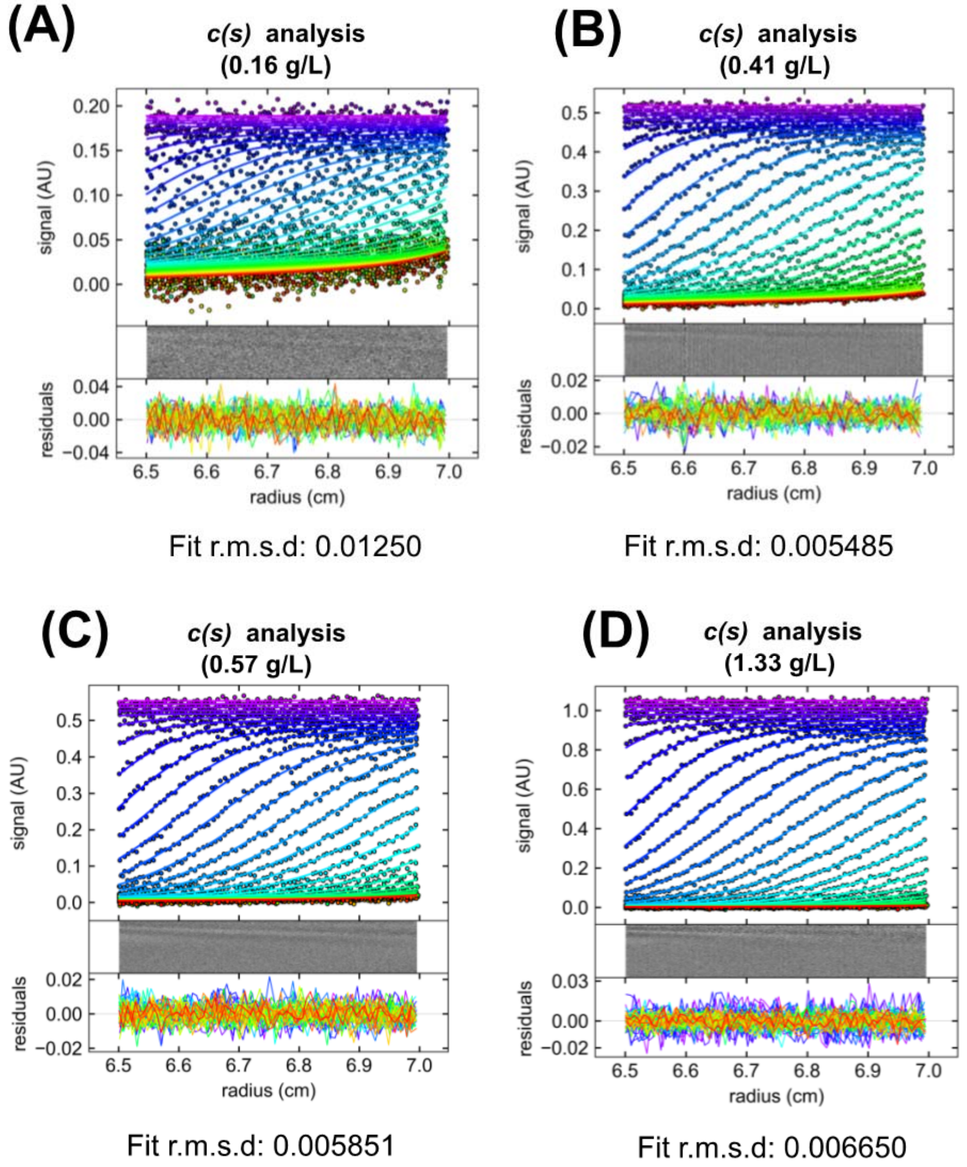

Supplementary figure 1. (A-D) OAS2 c(s) analysis using SEDFIT showing data, fit and residuals at different concentrations (0.16 - 1.33 g/L) of protein.

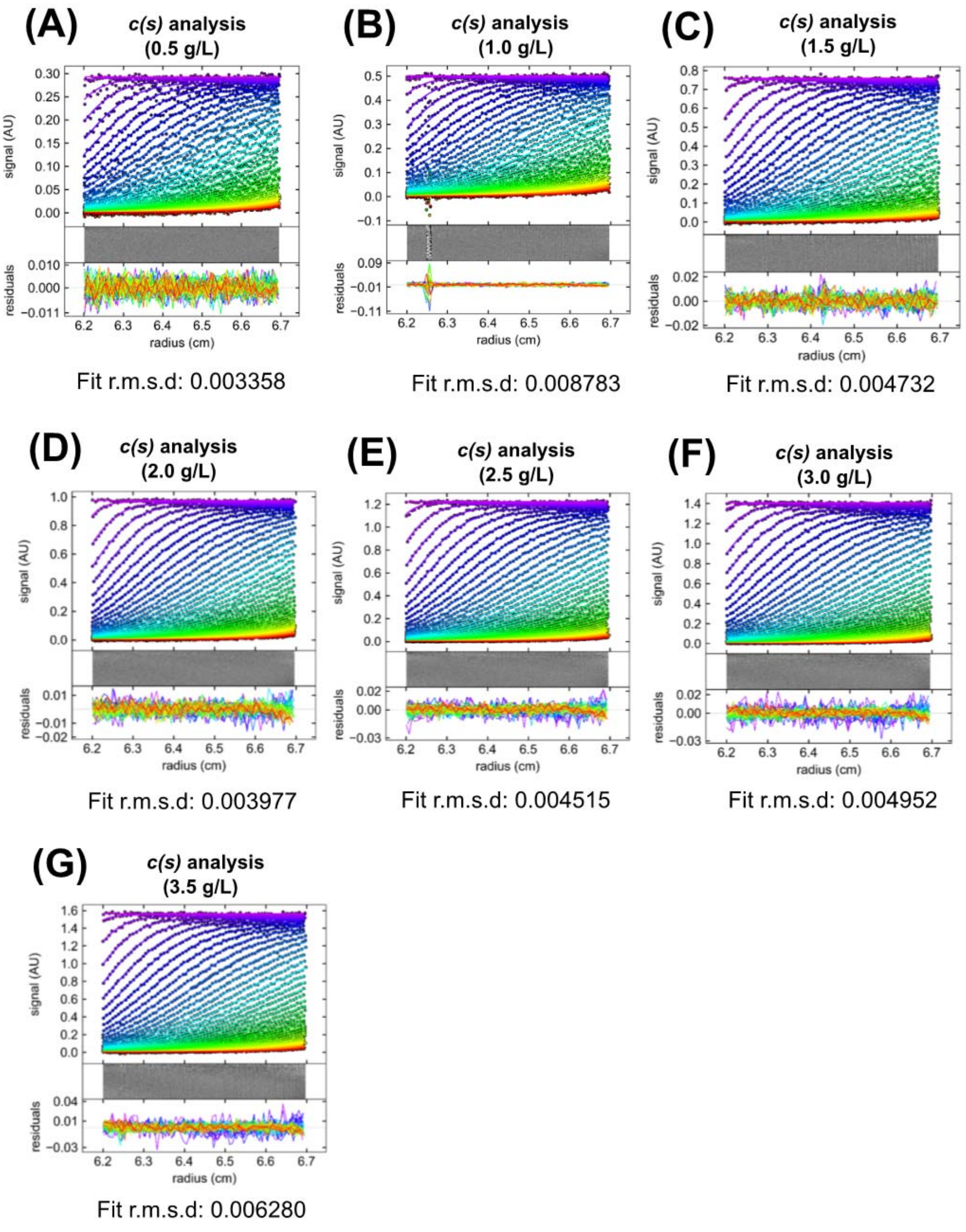

Supplementary figure 2. (A-G) OAS1 c(s) analysis using SEDFIT showing data, fit and residuals at different concentrations (0.5 - 3.5 g/L) of protein.

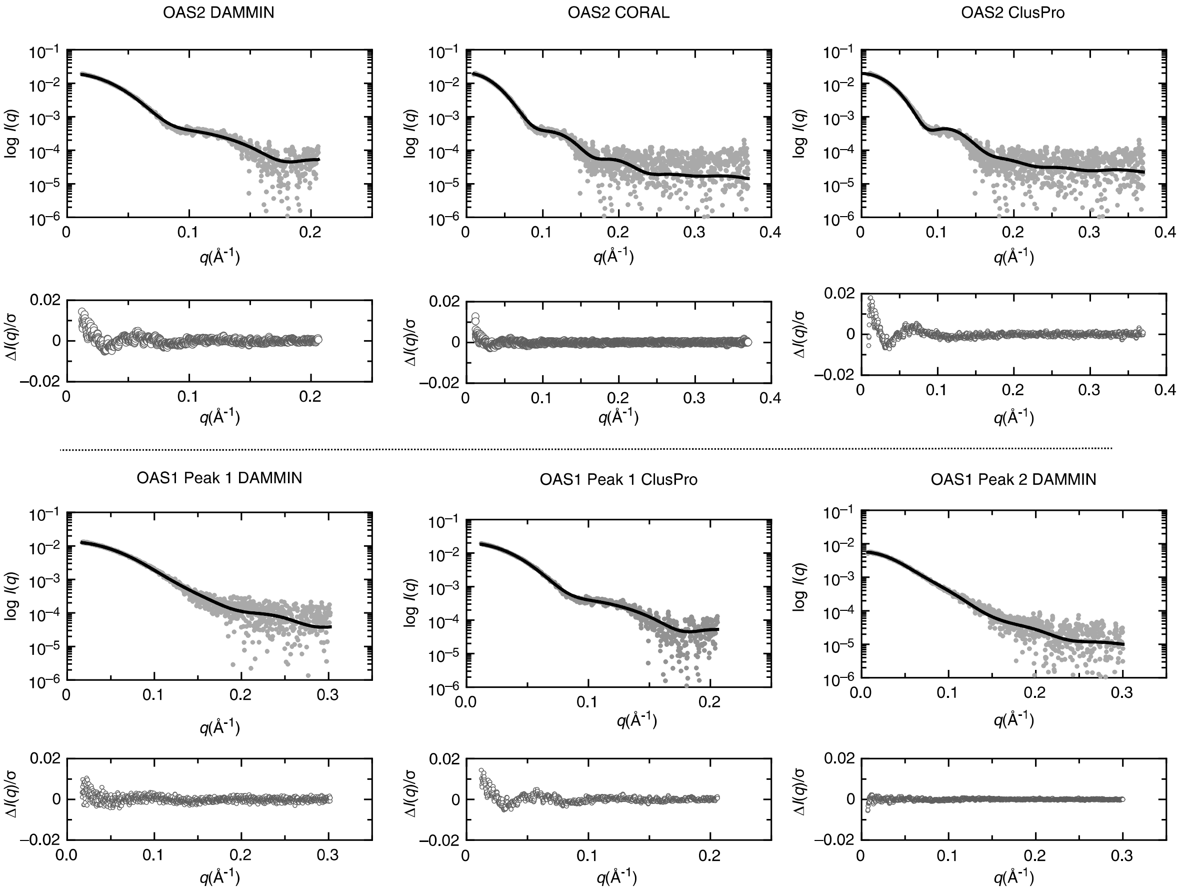

Supplementary figure 3. Alignment between experimentally collected SAXS data (dots in plots of log I(q) vs q^2) and calculated data from representative models (solid line in plots of log I(q) vs q^2). Plots of ΔI(q) vs σ representation residuals of alignment between experimentally collected SAXS data and data calculated from representative models.

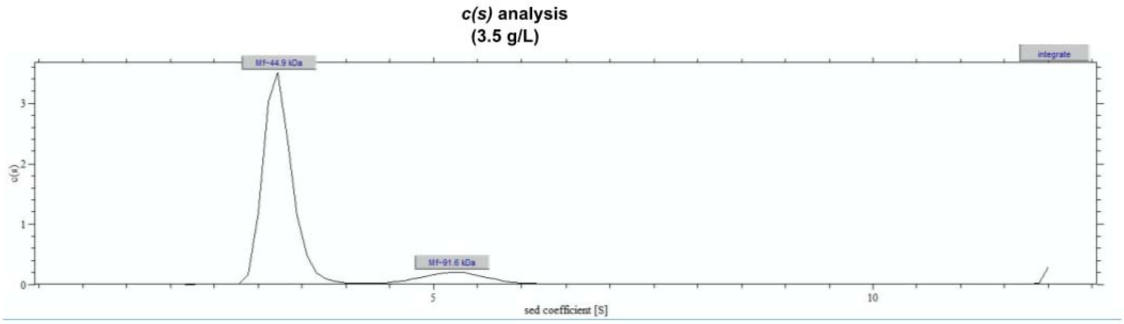

Supplementary figure 4. OAS1 c(s) analysis in SEDFIT showing molar mass of OAS1 monomer and dimer obtained using a single frictional ratio.
